# Influence of attitudes and behavior of milkers on the hygienic and sanitary quality of milk

**DOI:** 10.1371/journal.pone.0184640

**Published:** 2017-09-19

**Authors:** Oscar David Múnera-Bedoya, Laerte D. Cassoli, Paulo F. Machado, Mario Fernando Cerón-Muñoz

**Affiliations:** 1 Facultad de Ciencias Agrarias, Universidad de Antioquia, Medellín, Antioquia, Colombia; 2 Grupo de Investigación en Genética, Mejoramiento y Modelación Animal (GaMMA), Universidad de Antioquia, Medellín, Antioquia, Colombia; 3 Clínica do Leite, Universidade de São Paulo – ESALQ, Piracicaba, São Paulo, Brasil; Gaziosmanpasa Universitesi, TURKEY

## Abstract

Recognizing how human behaviors affect the milk process can be useful to understand variations in hygienic and sanitary parameters in bulk tank milk. Furthermore, this knowledge could be used to design management programs that guarantee milk quality, favoring the optimization of such processes. Forty-six milkers from the same number of dairy farms in Antioquia province (Colombia) were interviewed to establish the main factors associated to milk quality. Technical knowledge, motivations, and behavior of the personnel and its effect on hygienic and sanitary quality of milk were evaluated. Quality was assessed in terms of colony-forming units (CFU) and somatic cell count (SCC) in bulk tank milk. Two factors from a multivariate mixed data analysis were evaluated. One of those factors explained 9.51% of the total variability, related with in-farm availability and use of tools and the relationships between milker and manager. The other factor, associated with work environment and recognition, explained 6.97% of the total variability. The variables that best explained CFU levels were *Knowledge of the udder condition at milking*, and *Milking type (parlor or pasture)*. The SCC was associated to *knowledge of animal handling*, *schooling of milkers*, *milking site*, and the groups derived from the cluster analysis by farm. In conclusion, milker attitudes and behaviors can affect CFU and SCC in bulk tank milk.

## Introduction

Prices paid to dairy farmers worldwide increasingly depend on milk composition and quality. Composition depends on content of milk solids [1] while hygienic-sanitary quality is usually evaluated in terms of colony-forming units (CFU) and somatic cell counts (SCC). In Colombia, grams of fat and protein per litter as well as CFU levels determine the price paid to farmers [2], and efforts are on the way to include SCC [3].

Influence of mastitis and milking routines on milk quality is the subject of studies aimed to standardize milk production. Santana and Uribe [4] defined the milking routine as a strategy focusing on practices relevant to the milking process. Studies on these practices and their relationship with milk quality concluded that there exist other variables related to farm handling and management that should also be considered [5–7]. According to Reyes et al. [8], critical checkpoints must be established in the dairy farm and should be framed within a management program that includes a follow-up of mastitis, SCC, CFU, milking routines, and evaluation of milkers.

A successful implementation of handling practices aimed to control mastitis depends on attitudes and management style of the farmer. Increase of mastitis incidence can be related with sub-optimal farm management. Therefore, variations in parameters associated with SCC levels in bulk milk can be partially explained by attitudes of farmers toward mastitis prevention. The human factor represents the “farmer mentality" regarding milk quality [9].

Attitudes and motivations of farmers can influence performance parameters [9–11]. Behavioral theories have evaluated attitudes through questions on how good or bad the personnel are at carrying out specific tasks. Ajzen and Fishbein [12] and Ajzen [13] suggest that people with very positive attitudes regarding a task are more prone to practice it (theory of planned behavior). Jansen et al. [9], Jansen [14], and Lind et al. [15] applied these concepts to identify situational, personal and cognitive factors that explain why people carry out an action. The planned behavior theory describes the way certain behaviors are affected by individual factors (such as beliefs and attitudes), social factors (subjective norm), and perceptions (ways of communication, social pressure).

According to the planned behavior theory, the elements influencing a behavior are individual factors (behavioral attitudes), social factors (subjective norm), and the way the individual uses this information (perceived behavior control) to develop an activity (behavior) [13]). Good milking practices are the set of beliefs, values, attitudes and experiences that lead to good quality of milk [9, 12]. The objective of this study was to establish the influence of attitudes, behaviors, and milker´s knowledge on hygienic and sanitary parameters (CFU and SCC) related with bulk-tank milk quality.

## Materials and methods

This descriptive and exploratory work was conducted in two stages. On the first stage, a literature search established the state of the art and background of similar studies that applied behavioral theories in agriculture. In this stage, explicative variables for the theoretical constructs of the planned behavior theory by Ajzen [13] were identified and a measuring instrument was developed based on Jansen et al. [9]. On-site surveys to milkers assessed attitude criteria using likert-type questions, as recommended by Ajzen and Fishbein [12]. Negative or positive and favorable or unfavorable assessments were used to evaluate mentality, behavior and motivation of milkers (S1 Dataset) based on the work by Jansen et al. [9]. Surveys were conducted anonymously between January and December 2015. Respondents gave informed consent and authorized project personnel to visit the farms. The project did not present health risks to participants and involvement was voluntary. The survey was reviewed by a group of experts (veterinarians, agronomists, animal scientists and sociologists) and was validated with four farm employees under similar conditions. The information gathered was used exclusively for this study. The survey (S2 Dataset) included five sections in Part 1 "motivation of the milker", with information on "safety needs" (SN), "affiliation needs" (AN), "recognition and self-fulfillment needs" (RN) and "personal needs" (PN). Part 2 was related with "behaviors" (B), and Parts 3 and 4 with "mentality" (M) and "technical knowledge" (K), respectively. Finally, production and geographical information of farms was included.

On the second stage (simple cross-section) the problem was explained through a survey applied to 46 milkers selected at convenience in Bello, San Pedro de los Milagros, Belmira, Entrerríos, Donmatías, Santa Rosa de Osos, Yarumal, and San José de la Montaña municipalities. The last three municipalities formed a single group based on common characteristics, such as geographical proximity, agro-ecological conditions, technology and number of farms. The studied area is located as follows: latitude 6°17'14" and 7°14'31" North; longitude 75°11'45" and 75°44'03" West, corresponding to tropical lower montane wet forest. The genetic base is Holstein breed, grazing on Kikuyu pastures (*Cenchrus clandestinus* -Hochst. ex Chiov.- Morrone) and supplemented with concentrate at milking times (mechanical milking). Average cattle population is 45 animals per farm. The farms were included in the research project entitled "Productivity strengthening of the dairy chain in the Northern Antioquia district".

Milk quality information was obtained from bulk tank samples (8 mL) following protocols by the Milk Quality and Safety Laboratory at Universidad de Antioquia, accredited under standard NTC-ISO/IEC 17025:2005. The SCC Analyses were conducted in a CombiFoss Plus MilkoScan equipment (Foss, Denmark) based on cytometric flow, previously calibrated with standard raw milk (Eastern Laboratory Services, Medina, OH, USA). The CFU analyses were performed in a Bactoscan instrument (Foss, Denmark) through cytometric flow.

Average CFU and SCC were measured five months prior to the survey. Quality ranks for CFU (1000 units/mL) were based on Colombian regulations [3], as follows: excellent (< 75 units/mL), good (between 75 and 150 units/mL), acceptable (between 150 and 250 units/mL), and bad (> 250 units/mL). Ranks for SCC (1000 cells/mL) were: excellent (< 150 cells/mL), good (between 150 and 250 cells/mL), acceptable (between 250 and 400 cells/mL), and bad (> 400 cells/mL).

### Statistical analysis

The data set included 66 variables grouped in five survey sections, as follows: motivation, behavior, mentality, technical knowledge, and farm productivity and geographical information. Descriptive analyses were used to evaluate variability of the traits (traits that did not present variability were not included in subsequent analyses).

A factor analysis for mixed data was used to reduce dimensionality of the original database. The analyses allowed identifying the principal factors, which better describe the original dataset. Principal factors were analyzed according with the psychological constructs described in the planned behavior theory (behavioral attitudes, subjective norm, and perceived behavior control).

The principal factors were organized through hierarchical clustering on principal components [16]. This multivariate methodology allows studying similarities between individuals with respect to new variables (factors) obtained in the factor analysis for mixed data. Euclidean distances were used to calculate dissimilarities between observations. The Ward’s method was the character string used to define the clustering method. A category analysis was conducted with the information from the hierarchical analysis for variables CFU and SCC.

The factor analysis for mixed data was conducted with FAMD [17], while HCPC [18] was used for the hierarchical clustering analysis (significance at 0.05) following FactoMineR library procedures [17] of R-project [19].

## Results and discussion

The CFU and SCC results are shown in Table 1.

**Table 1:** Quality of bulk tank milk according to colony-forming units (CFU) and somatic cell counts (SCC).

Classification	CFU		SCC	
	Range (1000 units/mL)	n	Range (1000 cells/mL)	n
Excellent	< 75	28	< 150	6
Good	75–150	2	150–250	21
Acceptable	150–250	0	250–400	14
Bad	>250	16	> 400	5

Adapted from the Colombian Ministry of Social Protection [2] and the Ministry of Agriculture and Rural Development [3]. CFU: colony-forming units. SCC: somatic cells count. n: number of farms

The average number of cows in milking per farm was 44.98±20.78, with a milk production of 657.39±520.31 L/farm/day. Average milkers per parlor were 1.65±0.77, with median and average age of 28 and 30.7±9.62 years, and median and average job experience of 2 and 4.5±5.4 years, respectively. Regarding schooling, nearly 60% of the employees completed elementary school (5 years) and only half could read and/or write. Only 34.78% completed middle school and 6.52% finished high school (up to 12th grade). An overview of the farms is presented in Table 2.

**Table 2:** Milk quality, herd size and personnel characteristics of farms in Northern Antioquia*.

Municipality	n	CFU (000 units/mL)	SCC (000 cells/ mL)	Animals in production	Number of milkers	Age of milkers (years)	Seniority (years)
Bello	10	1174.52±2697.62	263.78±107.09	45.6±20.80	1.70±0.67	33.20±11.39	2.67±2.47
Belmira	3	5.43±3.44	218.07±118.28	60.00±19.70	2.67±1.53	32.00±2.00	8.33±10.12
Donmatías	3	142.94±228.38	224.08±32.21	42.00±9.85	1.67±0.58	37.00±6.08	4.11±4.44
Entrerríos	14	240.19±360.18	242.13±128.78	44.36±19.13	1.5±0.52	27.86±7.36	5.02±5.60
San Pedro	13	202.01±188.78	294.72±117.50	44.39±25.66	1.54±0.88	31.08±12.31	5.05±6.42
Other	3	143.73±234.23	199.72±36.56	36.33±22.03	1.67±0.58	26.33±4.04	2.33±2.31
Total	46	395.39±1296.88	256.30±111.20	44.98±20.78	1.65±0.77	30.70±9.62	4.50±5.40

*Mean ± SD. Significance level (p<0.05). CFU: colony-forming units. SCC: somatic cell counts. n: number of farms

Median and average CFU (assessed five months prior to the survey) were 283.00 and 395.39±1296.88 thousand units/mL, respectively, and 16, 2 and 28 farms ranked bad, good, and excellent milk quality, respectively. Median and average SCC were 239.3 and 256.0±111.2 thousand cells/ml, respectively, with 5, 14, 21 and 6 farms ranking bad, acceptable, good, and excellent, respectively.

Tables 3 to 6 present the five groups of variables included in the surveys. Descriptive parameters for variables related to "motivation of the milker", which included "safety needs" (SN), "affiliation needs" (AN), "recognition and self-fulfillment needs" (RN) and "personal needs" (PN), are presented in Table 3.

**Table 3:** Motivational variables for milkers in Northern Antioquia.

Variable	Mean (0–5)	CFU	SCC
**Safety Needs**			
Safety in the workplace is guaranteed (design of facilities and availability of protection implements)	4.63±0.9		
Employees have work stability at the farm.	4.85±0.63		
All required tools and equipment are always available for the job.	4.89±0.38		
People at the farm are treated with respect.	4.99±0.14		*
The family needs of milkers are covered (food, clothing, health, education, recreation)	4.87±0.4		
**Affiliation needs**			
Employees see the farm as their own.	4.72±0.78	*	*
All employees are important members of the work team.	4.96±0.21		
There is appreciation among co-workers.	4.87±0.33	*	
There is a feeling of appreciation from the manager.	4.91±0.28		
**Recognition and self-fulfillment needs**			
Milkers enjoy their job.	4.83±0.68		
Milkers feel proud of their work	4.98±0.14		
It is important that milk processors recognize the good quality of milk produced at the farm.	4.96±0.29		*
It is important that coworkers acknowledge good workers.	4.96±0.2		
It is important that managers acknowledge good employees.	4.89±0.52		*
Bad quality of milk is a concern.	4.85±0.47		
Milk quality is milker´s responsibility.	4.85±0.47		
The manager acknowledges a good work.	4.78±0.72		
The job offers learning opportunities.	4.76±0.84		
The manager takes milker´s ideas into consideration.	4.86±1.07		
Milker´s ideas are considered and implemented.	4.65±0.92		*
**Personal needs**			
Housing is cozy, with running water and electricity	1 ^1^		
The job allows enough resting time	0.85 ^1^		
There is always breakfast, lunch and dinner at the farm	1 ^1^		
Salary always arrives on time	1 ^1^		

* Significance level (p<0.05).

^1^ Ratio of affirmative answers for the variable.

**Table 4:** Behavior variables of milkers in Northern Antioquia.

Variable	Mean (0–5)	CFU	SCC
CMT is conducted at least twice a month.	4.46±0.94		
Milk samples from cows with recurrent mastitis are sent to the laboratory for bacterial cultivation.	4.39±1.16		
Gloves are always available for milking.	4.5±1.28		*
Elements for pre and post-milking teat dipping are always available	4.91±0.59		*
Milking equipment undergoes regular maintenance according to technical recommendations.	4.96±0.21		
Medicines for mastitis treatment are always available	4.91±0.46		*
Mastitis treatments follow the recommended dosage and frequency.	5±0		
Cow paths are kept in good condition.	4.63±0.85		*
Paper for teat cleansing is always available.	4.91±0.35		
Problematic animals (with recurrent mastitis) are culled.	4.78±0.73	*	*
Medicines are always available to treat dry cows.	4.98±0.15		
Employees undergo regular training for the job.	4.59±1		

* Significance level (p<0.05).

**Table 5:** Variables related to mentality of milkers in farms with mechanical milking systems in Northern Antioquia.

Variable	Mean	CFU	SCC
Mastitis is a concern for the milker.	4.78±0.69		*
Keeping mastitis low is important for milk production.	4.72±0.91		
The effort to keep low SCC is hard and the benefits are few.	2.46±1.76		
It is important to inform whenever a high productive cow has mastitis.	4.52±1.21		
Low SCC only benefits the milk processor (milk buyer)	2.32±1.75		
Antibiotics work well for mastitis.	4.3±1.26		
It is better to take action against mastitis only when advised by the the milk processor.	2.37±1.87		
When technicians say there is a mastitis problem their intention is selling mastitis treatments.	3.1±1.77		*
SCC reported by milk processors are unreliable.	3.91±1.64		
It is impossible to control mastitis	2.02±1.5		

* Significance level (p<0.05).

**Table 6:** Technical knowledge associated with milk quality and milking routines of milkers operating mechanical milking systems in Northern Antioquia.

Variable	Frequency of compliance (%)	CFU	SCC
Milker knows the proper condition of the udder at milking.	78	*	
Milker knows the proper condition of the cow tail at milking	91		
Milker knows how cows should be walked to the milking site.	98		
Milker knows the importance of milk 'stripping' into a black background.	72		
Milker knows how to handle very dirty cows at milking.	52		
Milker knows what to use to dry the udder.	72		
Milker knows how to attach the teatcups.	67		
Milker knows the post-milking routine.	98		
Milker knows the post-milking dipping process.	74		
Milker has a good relationship with his boss.	98		
Milker´s boss has a good attitude towards the milker.	96		

* Significance level (p<0.05).

The variables related with AN (housing, food and income) were excluded because all employees had maximum levels of satisfaction. Within the social dynamics of milk production in Colombia farm owners are responsible for providing these needs to employees as compensation for their work, and it is not part of the salary. In the light of the planned behavior theory these aspects present a favorable contribution within the subjective norm (social normative behavior) as long as they are met, based on which we can infer it positively stimulates developing behaviors associated with BMP in those farms.

Descriptive parameters for variables related with "behavior" (B) are presented in Table 4, while "mentality" (M) and "technical knowledge" (K) are presented in Tables 5 and 6, respectively.

The first two factors from the mixed-data factor analysis explained 16.48% of the total variance, with own values of 7.13 and 5.22. Factor 1 was related to availability and use of tools and occupational well-being, which explained 9.51% of the total variance. Variables with the highest contribution in the factor were associated with availability of tools and milking implements (B. *regular maintenance of milking equipment*, B. *availability of medicines to treat mastitis*, and SN, *availability of means to avoid exposing physical integrity*) and occupational well-being (SN. *respectful relationships*, RN. *recognition of good employees*, SN. *coverage of basic needs*, and PN. *sense of belonging*). The correlations of variables within the factor were positive.

Variables grouped in Factor 1 are social principles favoring a normative (subjective norm) to develop positive intentions for a behavior regarding BMP and other tasks pertaining to the job. Availability of milking tools and implements motivates compliance of the task and constitutes a behavioral attitude that favors the application of knowledge.

Factor 2 explained 6.97% of the variability. Among the most informative variables were those related to farm location, PN (*appreciation by the manager*, and *appreciation by the colleagues)*, and those related to RN (*listening to milker´s ideas*, *recognition as a good worker by colleagues*, *implementation of milker´s idea*s). For this reason, the factor was associated with good work environment and recognition. These results were similar to the ones reported by Bigras-Poulin et al. [20] and Tarabla and Dodd [21] who related attitudes, values and socio-demographic profile of farmers with variables connected to agricultural performance.

We obtained three clusters (CL) from the analysis of hierarchical agglomeration for milkers. CL1 and CL2 included two farms each, and CL3 had 44 farms. The significant variables (p<0.05) for the clusters were related with K on walking of cows to the milking site, condition of cow tail and its importance, teatcups attachment process, aside from SCC variables and resting time allowed in the job. Out of the 43 quantitative variables included in the analysis, 19 were significant (Table 7).

**Table 7:** General and cluster mean values for significant quantitative variables (p <0.05).

Quantitative Variable	Overall mean	Mean in Cluster 1	Mean in Cluster 2	Mean in Cluster 3
B. The milking equipment undergoes regular maintenance according to technical recommendations.	9.36E-16	-4.64		0.22
SN. Employees see the farm as their own.	-7.96E-17		-3.49	0.21
SN. Safety in the workplace is guaranteed (design of facilities and availability of protection elements)	3.16E-16		-3.47	0.20
RN. The manager takes milker´s ideas into consideration.	-3.64E-16		-3.36	0.19
SN. All required tools and equipment are always available for the job.	7.24E-17	-2.35	-2.35	0.22
B. Medicines are always available to treat dry cows.	-2.36E-15	-3.24		0.15
RN. It is important that the milk processors recognize the good quality of milk produced at the farm.	6.47E-16		-3.24	0.15
SN. People at the farm are treated with respect.	-2.36E-15	-3.24		0.15
SN. There is a feeling of appreciation from the manager.	1.39E-15	-3.20		0.14
RN. The manager acknowledges a good work.	1.18E-16	-1.76	-2.45	0.20
B. Medicines for mastitis treatment are always available	8.40E-16	-3.05		0.14
RN. Milker´s ideas are considered and implemented.	-2.32E-16		-2.87	0.17
AN. There is appreciation among co-workers.	-9.07E-16	-2.55		0.10
RN. It is important that coworkers acknowledge good workers.	9.36E-16	-2.21		0.09
AN. All employees are important members of the work team.	9.36E-16	-2.21		0.09
M. It is impossible to control mastitis	-5.92E-17		1.99	-0.09
RN. Milk quality is milker´s responsibility.	5.08E-16	-1.80		
M. Low SCC only benefits the milk processor (milk buyer)	-5.25E-17		1.53	-0.10
The effort to keep low SCC is hard and the benefits are few.	9.65E-17		1.45	-0.10

We found that K was highly qualifying, especially in tasks associated with milking routines. Although milkers correctly developed milking routines, they ignored the importance of some milking tasks related to udder health, animal comfort, and bulk milk quality. Ample knowledge generates a positive impact on the behavior of milkers facing activities to improve milk quality, aside from offering the opportunity of implementing control and evaluation of critical points in the process, as well as intervening whenever quality parameters are not optimum.

Variables related to RN, M -which were qualifying- directly influence the attitude toward behaviors associated with the milking process and milk quality. Regarding social environment of the milker, SNs associated with work stability and sense of belonging stood out. These variables, classified within the normative behavior (subjective norm) influence the intentions to improve milk quality.

Cluster 1 (CL1) included two farms with milking in parlor and excellent CFU values. The SCC was low in one of those two farms, and the other was high. The mean levels of significative variables for CL1 were negative (non-complying) especially for variables *(B) The milking equipment undergoes regular maintenance according to technical recommendations (-4*.*64)*, *(B) Medicines are always available to treat dry cows (-3*.*24)*, *(SN) People at the farm are treated with respect (-3*.*24)*, and *(SN) There is a feeling of appreciation from the manager (-3*.*20)* (Table 7).

Regarding CL2, this cluster grouped farms with non-complying parameters for all significant qualitative variables (Table 8). Non-complying levels were observed for variables *(SN) Employees see the farm as their own (-3*.*48)*, *(SN) Safety in the workplace is guaranteed (-3*.*47)*, *(RN) The manager takes milker´s ideas into consideration (-3*.*36)*, and *(RN) It is important that the milk processor recognizes the good quality of milk produced at the farm (-3*.*24)* (Table 7).

**Table 8:** Participation (%) of milkers in variables describing the clusters (p <0.05).

Categorical variables	Cluster 1	Cluster 2	Cluster 3
Milker doesn't know how to conduct cows to the milking site		100 (50)	
Milker knows how to conduct cows to the milking site		2.22 (50)	
Milker knows the proper condition of the tail tip during milking			95.24 (95.24)
Milker doesn't know the proper condition of the tail tip during milking			50 (4.76)

Value in parenthesis indicates the total percentage of milkers that meet the characteristic.

In CL3 several variables showed good results, especially *(K) Condition of the cow tail and its importance*, and included 95.24% of the farms (Table 8). Complying results were observed for variables *(B) The milking equipment undergoes regular maintenance according to technical recommendations (0*.*21)*, *(SN) Employees see the farm as their own (0*.*21)*, *(SN) Safety in the workplace is guaranteed (0*.*20)*, and *(B) Gloves are always available for the milking process (0*.*20)* (Table 7).

For CFU groups, out of the four categories (Table 1), K (*udder condition at milking*, *age of the milker*, *relationship between milker and manager*, and *milking site*) was significant (p<0.05).

Keeping the udder clean has been associated with hygienic quality of milk, environmental mastitis prevention and implementation of good farming practices. According to Elmoslemany et al. [22], udder hygiene, water temperature, and equipment cleansing products must be prioritized for proper CFU control. Future training efforts should focus on this aspect to improve the intention of implementing udder cleaning behaviors and care prior to milking as a control strategy of perceived behavior [9, 13]. Udder cleaning is paramount to keep low SCC and CFU levels. Calderón and Rodríguez [23] associated infectious mastitis with dirty bedding and udders.

The CFU levels were not very high; 65% for farms fluctuating between good and excellent CFU (4 and 61%, respectively), with levels lower than 150 thousand units/mL. This could be due to the training sessions on good milking practices that took place in the region in the past years. In that region, Ramírez et al. [5] found 50% of cows disinfected with iodized products after milking, 7.1% used other active principles, and 42.9% did not use post-milking dipping products.

For farms with bad CFU levels (>250 thousand units/mL), K (*handling of dirty udder*) and *milking site* were significant. 45.83% of milkers within this group have knowledge on udder handling prior to milking and 28.57% of farms in this group perform mechanical milking in parlor. Within these farms, none had excellent SCC levels. Milkers in these farms had higher than average satisfaction of needs but their ideas were not considered and felt they were not acquiring new learning, generating low affiliation feelings resulting from distant relationships with the employer. These milkers should have a training program and opportunities to gain new knowledge that allow them to intervene in the production process. This would -aside from normative behavior- increase the perceived control of their tasks, strengthen the learning process and improve the social environment in those farms.

Regarding the good-CFU category, significant variables (p<0.05) were *the relationship with the manager* (excellent and good), *performing of CMT at least once a month*, *SN (availability of tools for the job)* and PN (*sense of belonging*). In this group, 12.5% of milkers expressed good relationships with the manager, although none considered it excellent. For this group of milkers, B (*performing of CMT at least once a month*, *availability of elements for milking tasks*, and *sense of belonging*) was lower than average, leading to lower degree of satisfaction. These results could have caused low motivation to develop activities directed to gain control and follow-up of milking tasks, thus hindering excellent CFU.

Farms with excellent CFU had more positive valuations associated with satisfaction, indicating high motivation and constructive attitudes toward developing behaviors resulting in quality milk, among which is CMT implementation. This farm group had significant (p<0.05) variables associated to *relationship with the manager* (excellent) and *milking sites*, RN (*learning opportunities*), B (*performing CMT at least once a month*), PN (*sense of appreciation by the manager*, and *age of the milker* -with younger milkers in comparison to other farms). Age of milkers in this group was lower compared with the average (27.93 and 30.70 years of age, respectively). A similar result was reported by Lind et al. [15].

Regarding SCC groups (Table 1), the following were significant (p<0.05): K (*walking of cows to the milking site*, *farm cluster*, *milker schooling*, and *milking site*). There was a large effect of milker's attitude related to K, which contributed information on the behavior control of employees regarding milking tasks directed to quality milk.

As for the bad-SCC category, significant variables (p<0.05) were K (with 60% of milkers not knowing how teats should be dried), level 3 cluster (the one with the greatest number of farms), *age of milker* (very young milkers: 24.8 versus 30.7 years of age for all employees), M (*technicians talk about mastitis in order to sell treatments*, *mastitis is a constant concern*, and *keeping mastitis low is important for milk production*). Agreement or disagreement reported by milkers was low in all cases for significant variables (p<0.05), among them are B (*maintenance of milking equipment*, *availability of treatments for dry cows*, *availability of pre and post sealing of teats*, and *availability of gloves for milking*), M (*mastitis as a concern*, *technicians talk about mastitis for selling products*, and *keeping mastitis low is very important for milk production*), PN (*sense of belonging*, and *feeling as an important part of the team*), RN (*manager recognition*, *consideration* and *implementation of ideas*, and *recognition of milk quality by the processor*), and SN (*availability of means to prevent safety risks*, *respectful treatment*, and *coverage of family needs*).

The Acceptable-SCC category had significant variables for *number of milkers*, with 1.93 employees per farm (higher than the average, which was 1.65), and K (*proper maintenance of cow's paths)*, which was lower than the average.

Regarding teat drying, a study on mastitis prevalence in the area by Ramírez et al. [5] reported that only 3.6% of farms washed the udder prior to milking, 42.9% washed and dried it, and a 57.1% did not perform any cleaning procedure, which is associated to high incidence of mastitis. Low availability of elements for the milking routine and low equipment maintenance could be due to limited knowledge on good milking practices, among which is an adequate post-milking dipping procedure. Milkers showed lower SN and PN satisfaction compared with other SCC categories, which could have caused lower sense of belonging towards the farm and less motivation and attitude towards complying the activities associated with quality milk.

Geographic effect of farm resulted significant for the good-SCC group. The location effect stood out. Farms grouped at a greater distance from the milk processor were less technified, which has an effect on the normative behavior of milkers toward positive intentions to properly carry out their tasks for optimum SCC (farms located in San José de la Montaña, Yarumal, Santa Rosa de Osos and Donmatías). All farms having milking on pasture with vacuum line and 57% of farms with parlor milking systems were in this group.

The variables related to M were significant (*reporting cows with mastitis*, *keeping SCC low requires a lot of effort but benefits are few*, *impossibility to control mastitis*, and *technicians talk about mastitis to sell products*) with results below the average. The B variable (*availability of gloves for milking*) was higher than average, just as PN (*sense of belonging*). Levels lower than the average were also reported for RN *consideration and implementation of ideas*, and *sense of appreciation by colleagues as a good worker*.

Finally, the excellent-SCC category presented significant variables for K, with higher valuations for *how should teatcups be attached*, *how should the post-milking dipping be performed* (p<0.05), and *how should cows be walked to the milking site* (p = 0.08). Regarding schooling, 100% of milkers who had graduated from high school were in this group, as well as 33% of employees who could only read and/or write. None of the farms with bad-CFU were part of this category. High knowledge and schooling levels favored milk quality.

Employees in this category (with 2.8 years of age difference with respect to the total) had better attitudes, knowledge and more perceived behavior control. Strategies should be developed to reduce the gap between scientific knowledge on mastitis and its assimilation and application by farmers [5]. Application of these practices alone does not guarantee good health of the udder; they are just part of a number of management factors such as good animal, nutrition, hand washing prior to milking, avoiding cow stress at milking, having equipment properly maintained, using appropriate vacuum pressure to avoid trauma or deficient milking, among others.

In agreement with Valeeva et al. [24], we found important internal and external factors related with farmer motivations and its association with mastitis incidence. Factors that motivate farmers to adopt recommended practices should be identified in order to reduce SCC. Internal factors of the farmer (individual) provide more motivation than external factors (i.e., knowledge about performance of the entire dairy sector).

## Conclusions

This study shows that some variables can be associated with milker´s intentions toward developing behaviors related to milk quality parameters, expressed as CFU and SCC in bulk tank. The survey to milkers shows the influence of a subjective or social norm mostly associated with availability of tools and relationships between milker and manager, followed by milker´s attitudes toward behaviors associated with milk quality and his degree of self-fulfillment. Furthermore, the knowledge of milkers on quality milk process and average SCC in tank are qualifying for dairy farms.

Improvement of milk hygienic and sanitary quality is influenced by the milker's attitude toward behaviors associated with the milking routine, aside from knowledge on cleanliness of the mammary gland prior to milking and proper conduction of cows to the milking site. Therefore, attitudes and knowledge should be considered in research and promotion programs aimed at improving milk quality parameters, as well as training programs on good milking practices.

This study contributes to empirical research on the social processes applicable to the study of milk quality and is a good starting point for future research on the subject. Additionally, it establishes the basis to develop strategies for controlling quality parameters of bulk tank milk, which include aspects associated with attitudes, knowledge and behaviors of the personnel involved in the milking process.

## Supporting information

S1 Dataset This file contains the database used for the analysis presented in this work. (CSV)

S2 Dataset This file contains the survey questions used in the study, in both the original language and English. (XLSX)
